# Sugary snack restriction enhances body composition improvement in overweight women engaging in non-face-to-face walking during COVID-19

**DOI:** 10.3389/fpubh.2024.1396598

**Published:** 2024-06-03

**Authors:** Youngjun Lee, Nahyun Kim, Seunghwan Go, Jisu Kim, Jonghoon Park

**Affiliations:** ^1^Physical Activity in Youth with Disabilities Laboratory, Department of Kinesiology, Michigan State University, East Lansing, MI, United States; ^2^Exercise Nutrition and Biochemistry Laboratory, Department of Physical Education, Korea University, Seoul, Republic of Korea; ^3^Department of Sports Medicine and Science in Graduate School, Konkuk University, Seoul, Republic of Korea; ^4^Physical Activity and Performance Institute, Konkuk University, Seoul, Republic of Korea

**Keywords:** sugar, exercise, walking, body composition, glucose metabolism, overweight, young women, COVID-19

## Abstract

**Introduction:**

This study assesses the impact of dietary modification, specifically sugary snack restriction, in conjunction with a brisk walking program on overweight management in young overweight women, with a focus on changes in body composition and glucose metabolism.

**Method:**

An 8-week randomized controlled trial was conducted amidst the COVID-19 pandemic, utilizing a remote intervention approach to comply with health guidelines and ensure participant safety. The study’s remote nature highlights adaptability in health interventions during challenging periods, such as the COVID-19 pandemic. Twenty-one overweight Korean women aged 20–39, with an average BMI of 24.6, were selected for the study. They were divided into two groups: one engaging in brisk walking and the other combining this exercise with a sugary snack restriction, demonstrating the study’s focus on comparative intervention efficacy.

**Results:**

The exercise-only group showed notable reductions in glucose, insulin, HOMA-IR (*p* < 0.05), and total cholesterol levels (*p* < 0.01). In contrast, the group that combined exercise with dietary modification displayed more pronounced improvements in body weight, fat mass, and waist circumference (*p* < 0.05). This differential outcome emphasizes the added benefit of integrating dietary control with physical activity.

**Discussion:**

The findings suggest that adding a dietary component, particularly a sugary snack restriction, to an exercise regimen can significantly enhance the effectiveness of overweight management in young women. This study underscores the importance of holistic lifestyle interventions that combine dietary and physical activity modifications for improved health outcomes.

## 1 Introduction

Household configurations are experiencing rapid transformations attributed to the erosion of traditional family structures, a growing prevalence of individuals opting for single or nuclear family arrangements, and societal shifts, including advancements in women’s economic empowerment (1). According to recent findings from Statistics Korea, the incidence of single-person households in Korea has almost doubled, surging from 15.5% in 2000 to 29.9% in 2019, establishing it as the predominant family structure in the nation. Furthermore, projections indicate that single-person households are poised to constitute 37.7% of the total by 2047, with the sharpest growth rate observed among countries within the Organization for Economic Co-operation and Development. Particularly noteworthy is the pronounced rise among young adults aged 20–39, with a substantial increase in the number of women residing in single-person households (2). Single-person households have been linked to reduced physical activity and unhealthy dietary habits, heightening the risk of obesity in comparison to multi-person households (3, 4). Research on the general adult population in Korea indicates that individuals living alone tend to consume a higher proportion of energy from fats and a lower intake from carbohydrates and plant proteins (5, 6). This dietary pattern is associated with an increased risk of metabolic syndrome and obesity (6). Notably, single-person households in metropolitan areas in Korea exhibited a higher tendency to skip breakfast and experience food insecurity, factors that are linked to an increased risk of metabolic abnormalities such as obesity (7). Moreover, single-person households represent a vulnerable demographic at an elevated risk of social isolation, exacerbated by infectious diseases such as COVID-19 and subsequent social distancing measures. Such isolation often correlates with higher levels of stress and a lack of motivation, which can lead to coping strategies that include the increased consumption of sugary snacks and beverages (8, 9). These dietary choices, driven by emotional responses rather than nutritional needs, further compound the risk of metabolic abnormalities and obesity. Under these circumstances, the implementation of non-face-to-face intervention programs becomes imperative to address the multifaceted contributors to overweight within single-person households.

Epidemiological evidence, coupled with plausible mechanisms and clinical data derived from dietary intervention studies, strongly substantiates a direct causative link between sugar intake and metabolic diseases (10). Particularly, sugar-rich snacks and beverages represent prominent sources of added sugars in our dietary patterns. In a cross-sectional study encompassing 1,487 adults, there was a positive correlation observed between the consumption of sugary snacks and both body mass index (BMI) and waist circumference (WC) (11). Additionally, among overweight individuals aged 20–50 years, a 10-week intervention featuring a high sucrose intake (comprising 28% of total energy consumption), primarily through beverages, resulted in increased energy intake, body weight, and fat mass (12). The adverse impact of sugar-rich beverages on the development of obesity may be attributed to their ability to induce rapid and substantial elevations in blood glucose and insulin levels (12). Conversely, substituting sugar-sweetened beverages with either artificially sweetened or unsweetened alternatives over 12 months led to a reduction in body weight among individuals with central adiposity (11). The greater weight and fat loss observed in response to a low-sugar diet could be linked to a decrease in the secretion of leptin, an appetite-suppressing hormone (11). This reduction in leptin levels stimulates osteoblasts in the bone through brain signaling, ultimately augmenting the secretion of uncarboxylated osteocalcin (ucOC), a hormone released by bone tissue that is associated with anti-diabetic and anti-obesity effects (13). Consequently, limiting the consumption of sugary beverages can assist individuals in adopting healthier dietary patterns and maintaining a healthy body weight (14, 15).

Brisk walking is a frequently adopted exercise regimen among adults aiming to control their body weight (16). In the case of overweight adults, engaging in brisk walking interventions carries a minimal risk of injury while proving highly effective in achieving weight loss and mitigating the risk of metabolic conditions, including diabetes (17, 18). A systematic review and meta-analysis have indicated that brisk walking can efficiently reduce body weight, waist circumference, and fat mass in individuals below 50 years of age who are dealing with obesity (19). Furthermore, moderate-intensity exercise has been demonstrated to stimulate the secretion of osteocalcin (OC), potentially contributing to an amelioration in serum glucose levels (20, 21).

The rationale for focusing on women specifically lies in the unique metabolic and hormonal differences that influence how they process, and store fat compared to men. Women tend to have a higher percentage of body fat and different fat distribution patterns, which are influenced by hormonal fluctuations related to the menstrual cycle, pregnancy, and menopause (22). These factors can affect energy balance and the risk of developing metabolic disorders. Furthermore, women, particularly those living alone, may experience higher level of stress and emotional eating, leading to increased consumption of sugary snacks and beverages as coping mechanisms (23). By addressing these specific needs, our study aims to provide tailored interventions that can more effectively support women in managing their weight and improving metabolic health. Previous research has highlighted the success of gender-specific approaches in dietary and exercise interventions, suggesting that such strategies can yield better outcomes by considering the unique physiological and psychological needs of women (24–26).

To the best of our understanding, there has been no prior exploration into whether implementing a management program focused on restricting sugar-rich snacks and beverages could augment the benefits of a brisk walking exercise regimen on metabolic outcomes within overweight populations. This study comes at a critical time when the demand and need for effective lifestyle programs are on the rise for overweight management in Korea, reflecting a growing public health concern (27). Consequently, this study aims to examine the impact of limiting sugar-rich snacks and beverages on glucose metabolism markers and body composition among young women who are overweight and engaging in a non-face-to-face brisk walking program during the COVID-19 pandemic.

## 2 Materials and methods

### 2.1 Subjects

We recruited participants through flyer advertisements and social media over 1 week from March 22 to March 28, 2021. Volunteers (*n* = 40) expressed willingness to participate in this study and were screened by the research investigators. The sample size of the study population was determined using power analysis with the G-power 3.1 program, setting the significance level at below 0.05, power at 80%, and effect size at 0.05. The inclusion criteria for the study are as follows: (1) Participants must be female; (2) They must be a Korean nationality; (3) They should be aged between 20 and 39 years; (4) Participants need to have a BMI of at least 23 kg/m^2^, categorizing them as overweight according to World Health Organization standards for Asian populations; (5) They must possess a body fat percentage of 30% or higher; (6) They should be capable of undergoing brisk walking. These criteria were chosen based on previous studies indicating higher health risks and potential benefits from interventions in these groups (28, 29). The exclusion criteria include: (1) Participants with menstrual irregularities or amenorrhea, to control for hormonal influences that could affect the study’s outcomes; (2) Those suffering from any systemic diseases like diabetes or chronic heart conditions, to avoid acute health fluctuations; (3) Those on medication for metabolic diseases such as insulin, to eliminate the effects of such medications on metabolic outcomes; (4) Participants who regularly engage in exercise, defined as more than 150 min of moderate exercise per week, to focus on less active individuals. After screening and application of inclusion and exclusion criteria, 24 eligible participants were selected for inclusion in the study. These participants were then allocated into one of two intervention groups using a stratified randomization approach to balance the groups based on key demographic and baseline health characteristics (age, height, BMI, and body fat percentage, WC). This randomization was facilitated by a computerized random number generator software, which assigned participants to either the exercise and control (ExC; *n* = 13) or exercise and nutrition (ExN; *n* = 11) groups. The use of this software ensured that the allocation process was unbiased and that the assignment to the intervention groups was completely random. Participant characteristics are shown in Table 1.

**Table 1:** Descriptive characteristics of the participants.

Variables	Exercise and control (*n* = 13)		Exercise and nutrition (*n* = 11)		*p*-value
	*M*	SD	*M*	SD	
Age (years)	28.0	4.2	30.4	6.3	0.295
Height (cm)	164.9	4.1	162.0	4.6	0.128
Body weight (kg)	66.8	10.2	61.9	10.3	0.259
BMI	24.6	3.8	23.6	3.9	0.532
WC (inch)	31.7	3.6	31.1	3.7	0.668

M, mean; SD, standard deviation; BMI, body mass index; WC, waist circumference.

### 2.2 Experimental design

**Figure 1:**
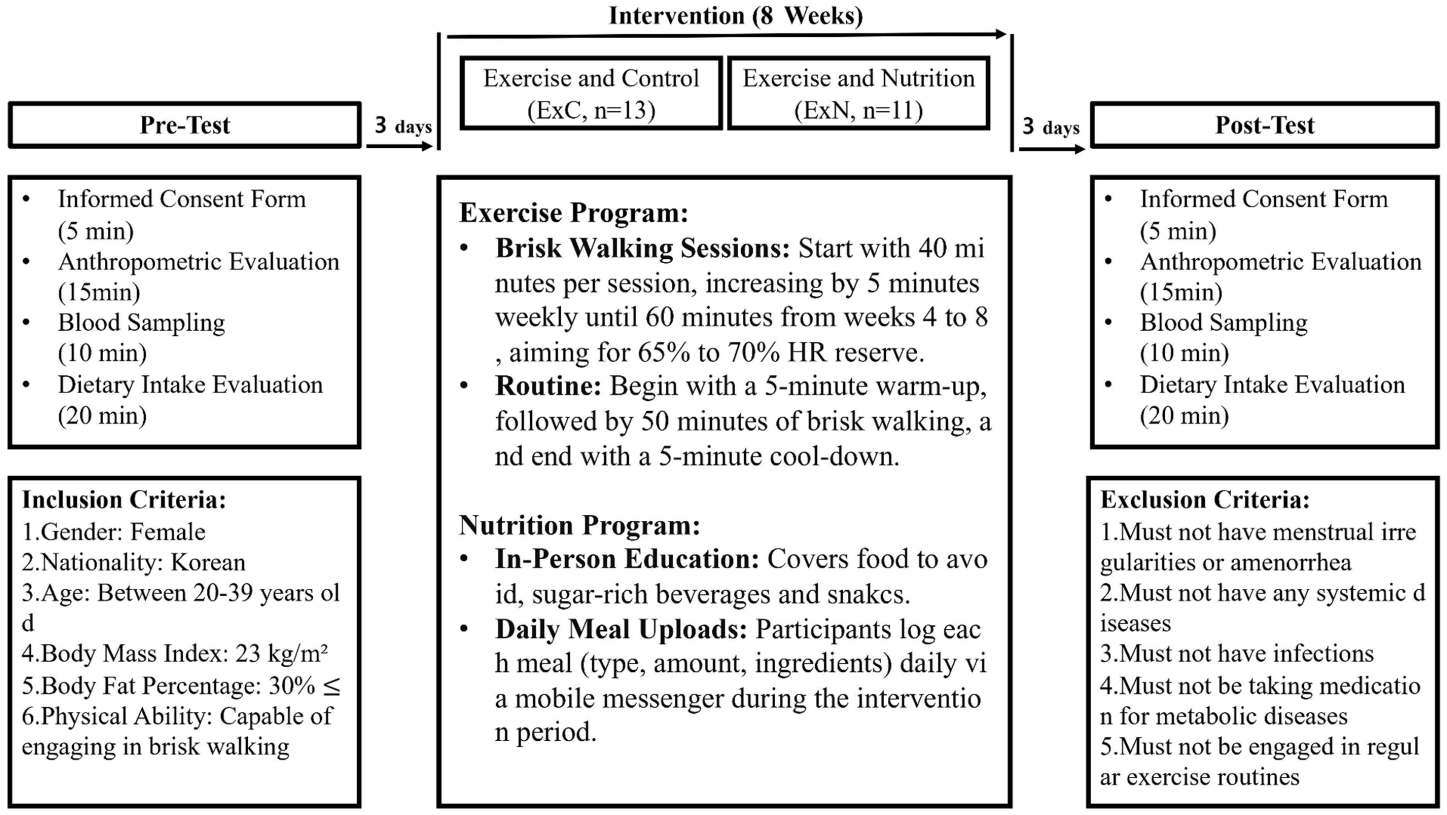
Comprehensive flowchart of the study design.

The study was a non-face-to-face eight-week intervention trial. ExC and ExN groups participated in the non-face-to-face behavioral program of brisk walking at four sessions per week over 8 weeks. The ExC group focused solely on the exercise component without additional dietary guidance. The subjects maintained other habitual or recreational physical activities, such as housework, during the study. The ExN group further received nutrition education about the harmfulness of excess sugar intake causing overweight one time before starting the intervention. The ExN group was managed through social media or messages from phones to limit their intake of sugar-rich snacks and beverages during the intervention period. The participants consumed their usual diet 3 days before the test, then fasted (except for water), and abstained from exercise for 10–16 h on the night before the test. All measurements were carried out in consideration of changes in hormones and biometric data according to a woman’s menstrual cycle. The experiment design involved a one-day pre-test session, 8 weeks of intervention, and a one-day post-test session. Post-intervention testing was completed >72 h after the final exercise session. During the intervention period, each participant visited the laboratory once every 2 weeks to gradually increase the exercise intensity of brisk walking. Dietary intake and body composition were measured at 4 weeks and 8 weeks (Figure 1).

### 2.3 Exercise program

Brisk walking was conducted at 65 to 70% of their heart rate (HR) reserve for 40 min per session with increments of 5 min in duration per week until week 4 and for a maximum of 60 min per session from weeks 4 to 8. Before starting the intervention, participants checked their intensity of brisk walking on the treadmill using an HR monitor (S810, Polar, Kempele, Finland) at the laboratory. After that, participants visited the laboratory every 2 weeks and checked the intensity on the treadmill using the HR monitor to reach the target of 65–70% of their HR reserve. When HR dropped below the target intensity, we increased the treadmill speed to ensure they reached the target HR. Usual walking training was conducted non-face-to-face with each subject. During the training period, they were instructed to begin each session with a 5-min gradual warm-up and walk briskly for the remainder of the session (50 min). After the walking sessions, they were instructed to conduct a 5-min cool-down. They were requested to use the smartwatch parameters (HR) per session and upload their exercise load (i.e., time, pace, and distance) through a mobile messenger. Records were checked every day by the researcher after which the participants were given feedback about the exercise load. The total amount of physical activity (excluding warm-up and cool-down) was computed as metabolic equivalents (METs). The rationale for the chosen intensity and duration is based on previous studies showing effective weight management at this level of activity (30, 31).

### 2.4 Nutritional education program

To address the dietary imbalances, our study implemented a comprehensive nutritional education program. It includes food information to avoid in various situations, beverages that contain large amounts of sugar, and alternative foods to sugary snacks. Participants were grouped into chat rooms using the messenger app, where they uploaded photos of all meals and snacks. These photos were analyzed using the Computer Aided Nutritional Analysis Program (CAN-Pro 5.0) to evaluate nutritional content. Based on the analysis, participants received feedback on their dietary quality and personalized recommendations on how to adjust their nutrient intake, especially for the sugar intake. To support sustained healthy eating habits, participants were given positive feedback regularly. The program ensured daily monitoring and tailored advice to help participants improve their dietary patterns effectively. The rationale for the nutritional education program is supported by research showing the effectiveness of tailored dietary interventions in reducing sugar intake and improving health outcomes (32).

### 2.5 Measurements

#### 2.5.1 Anthropometric evaluation

Height, and body composition (body weight, fat free mass and fat mass) were measured using bioelectrical impedance analysis equipment (Inbody 770; Inbody, Seoul, Korea). Waist circumference (WC) was measured midway between the lowest rib and the iliac crest in the standing position using a measuring tape.

#### 2.5.2 Blood biomarkers

Blood samples were centrifuged at 4,000 g for 10 min, and serum samples were stored at −80°C until analysis. Fasting serum glucose (FSG), triglyceride (TG), total cholesterol (TC), high-density lipoprotein cholesterol (HDL-C), fasting serum insulin, ALTSGPT, ALTSGOT, and uric acid were quantified by GC Labs (Gyeonggi-do, South Korea). Whole-body insulin resistance was estimated by the homeostasis model assessment of insulin resistance (HOMA-IR) index [FPI (mg/dl) × FSG (uIU/ml)/405]. Serum levels of cOC and ucOC were determined using a commercially available Gla-type osteocalcin (Gla-OC) EIA Kit (MK111; Takara Bio Inc., Tokyo, Japan) and Glu-type osteocalcin (Glu-OC) EIA Kit (MK118; Takara Bio Inc.), respectively. The total level of OC was calculated as the sum of cOC and ucOC.

#### 2.5.3 Dietary intake

Amounts of energy (i.e., total calorie, carbohydrate, fat, and protein intake) were collected through a 24-h recall. This 24-h recall was conducted twice: pre-and post-intervention. For each assessment, participants recalled their dietary intake for three specific days from the preceding week-two on weekdays and one on a weekend day to ensure a comprehensive evaluation of energy intake. During face-to-face interviews with trained researchers, models and pictures of foods and dishes were used to help participants assess the sizes of standard food portions. Questions about snacks, drinks, and supplements were also asked. Nutritional analyses were performed using the CAN-Pro 5.0. The use of multiple recall days and detailed face-to-face interviews enhances the accuracy of dietary intake assessment, as recommend in previous nutritional research (33).

#### 2.5.4 Physical activity

Records on the distance (km/day), time (minute/day), and speed (minute/km) of brisk walking during the 8-week intervention were calculated using a running record application called “Nike Run.” The records were presented by taking the mean up to 2, 4, and 8 weeks at intervals. The total energy expenditure was calculated using METS, which is a simple, practical, and easily understood procedure for quantifying the energy cost of activities. A value of 1 METS equals 3.5 mL/kg/min. Based on the collected data on distance, time, and speed of exercise, the intensity of exercise per session was converted into a METS value and substituted into the following formula. The choice of using METS and detailed tracking via mobile application is supported by research indicationg their reliability and effectiveness in monitoring physical activity (34, 35).

$$\mathrm{Energy}\;\mathrm{expenditure}=\mathrm{METS}\;\mathrm{value}\times \mathrm{time}\;\left(\mathrm{minute}\right)\times \mathrm{weight}\;\left(\mathrm{kg}\right)$$

### 2.6 Statistical analyses

All data were analyzed using Statistical Package for Social Sciences (SPSS 18.0 K; IBM, United States). Data were presented as mean ± SD. Paired *t*-test was used to examine differences in baseline and follow-up variables within each group. An unpaired *t*-test was used for comparison between the ExC group and the ExN group. For variables with non-normal distribution, the Mann–Whitney U-test was performed. Pearson’s correlation analyses were used to assess correlations among changes in anthropometric measurements and total OC, cOC, and ucOC. Simple linear regression models were used to assess the influences of changes in OCs on anthropometric measurements, including changes in BMI, WC, and percentage of body fat. Statistical significance was set at *p* < 0.05.

## 3 Results

### 3.1 Participant enrollment and attrition

During the first 4 weeks, one participant withdrew for personal reasons (business trips). Four weeks after the intervention started, two participants dropped out due to infection with COVID-19 or close contact. The final number of participants is 21. The data of 21 young women (mean age: 30.24 ± 5.09 years; BMI: 24.59 ± 3.75; % body fat: 35.31 ± 6.01; waist circumference [WC]: 31.84 ± 3.41 inch) were analyzed. No significant difference was found in all variables between the two groups at the baseline (Week 0).

### 3.2 Exercise and energy expenditure

**Figure 2:**
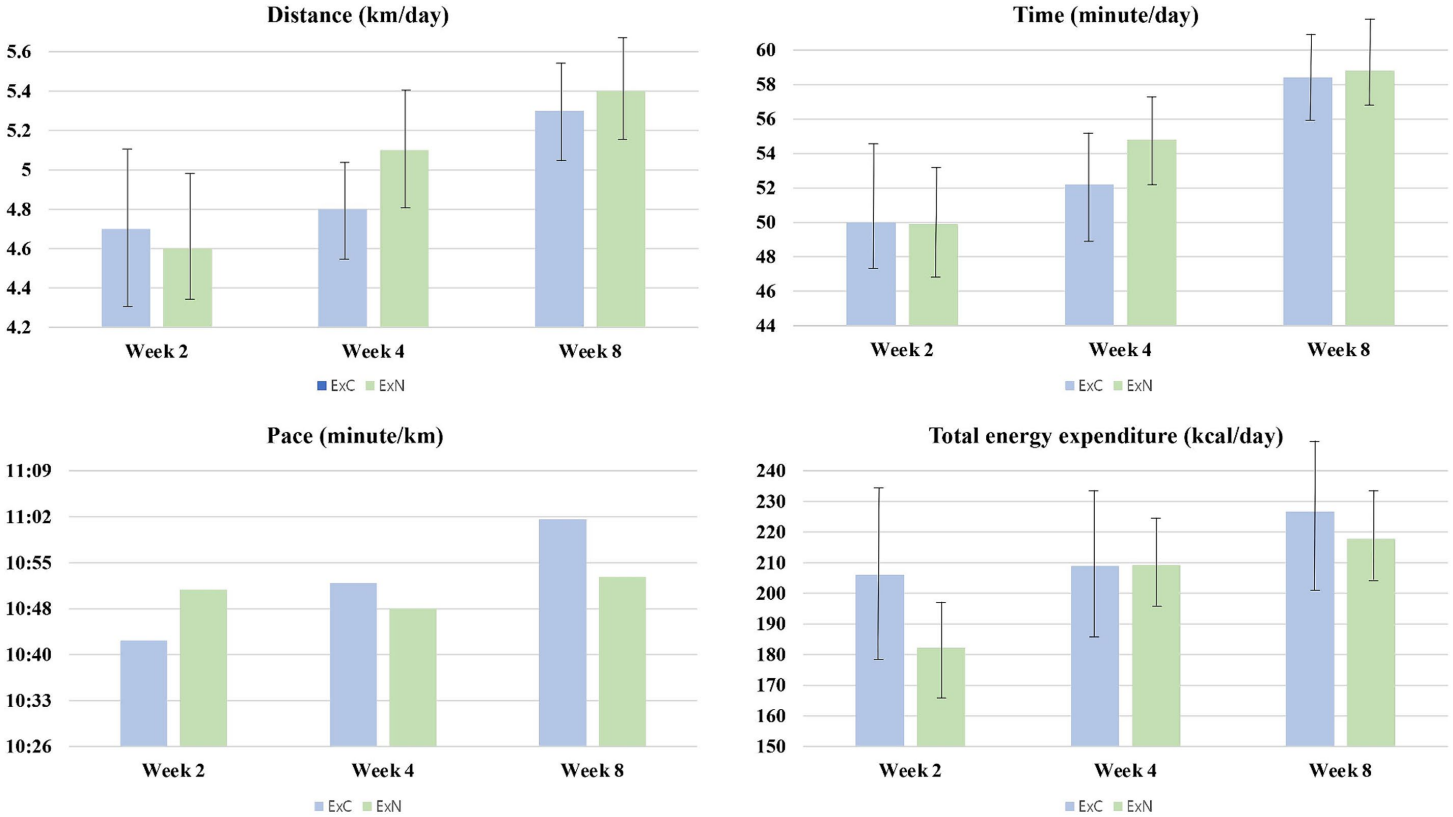
Trends in physical activity levels among participants during the experiment for ExC group and ExN group.

Table 2 presents the estimated energy expenditure of the participants during the eight-week intervention at weeks 2, 4, and 8. No significant difference was found for exercise in terms of distance, time, speed, and total energy expenditure between the two groups in weeks 2, 4, and 8. The average exercise distance across 8 weeks was 5.3 ± 0.5 km/day (ExC group) and 5.4 ± 0.5 km/day (ExN group); the average exercise time was 58.4 ± 4.7 min/day (ExC group) and 58.8 ± 4.8 min/day (ExN group); average speed was 11.02 min/km (ExC group) and 10.53 min/km (ExN group); lastly, total energy expenditure was 226.7 ± 50.8 kcal/day (ExC group) and 217.9 ± 31.9 kcal/day (ExN group) (Figure 2).

**Table 2:** The estimated energy expenditure of the participants.

Variables	Follow-up	Exercise and control (*n* = 10)		Exercise and nutrition (*n* = 11)		*p*-value
		*M*	SD	*M*	SD	
Distance (km/day)	Week 2	4.7	0.8	4.6	0.7	0.761
	Week 4	4.8	0.5	5.1	0.6	0.352
	Week 8	5.3	0.5	5.4	0.5	0.732
Time (minute/day)	Week 2	50.0	9.4	49.9	6.7	0.980
	Week 4	52.2	6.4	54.8	5.8	0.338
	Week 8	58.4	4.7	58.8	4.8	0.843
Pace (minute/km)	Week 2	10:43		10:51		0.520
	Week 4	10:52		10:48		0.815
	Week 8	11:02		10:53		0.995
Total energy expenditure (kcal/day)	Week 2	206.0	56.6	182.3	31.9	0.262
	Week 4	209.0	49.0	209.2	29.5	0.992
	Week 8	226.7	50.8	217.9	31.9	0.646

M, mean; SD, standard deviation.

### 3.3 Dietary intake and nutritional changes

Table 3 depicts energy intake during the eight-week intervention at weeks 2, 4, and 8. A significant reduction was observed in total energy intake (*d* = −377.6 ± 445.7) and carbohydrate intake (*d* = −301.4 ± 255.5) for the ExN group; however, we observed no significant differences for the ExC group.

**Table 3:** Baseline and follow-up energy intake of the subjects.

Variables	Exercise and control (*n* = 10) Age: 28.3 ± 4.3								Exercise and nutrition (*n* = 11) Age: 32.0 ± 5.3							
	Week 0		Week 4		Week 8		*d*		Week 0		Week 4		Week 8		*d*	
	*M*	SD	*M*	SD	*M*	SD	*M*	SD	*M*	SD	*M*	SD	*M*	SD	*M*	SD
Total energy intake (kcal/day)	1822.9	223.1	1464.7	466.9	1590.8	243.3	−232.1	403.2	1582.0	340.6	1139.2	366.7	1204.5	330.7	−377.6*	445.7
Carbohydrates intake (kcal/day)	923.6	156.3	688.0	238.8	802.2	216.7	−121.4	270.3	815.6	270.0	546.3	224.3	514.2	175.7	−301.4**	255.5
Fat intake (kcal/day)	579.3	90.2	494.9	252.7	463.4	228.1	−115.9	181.8	469.0	118.7	363.1	171.0	453.2	159.6	−15.8	199.7
Protein intake (kcal/day)	291.3	43.9	278.7	89.8	282.2	64.8	−9.1	72.4	264.9	78.4	252.3	66.5	247.0	65.8	−17.9	105.2

M, mean; SD, standard deviation. Week 0, before intervention; week 4, at the beginning of the 4th week of the intervention; week 8, after intervention; d: (week 8) – (week 0). P difference between baseline and follow-up values; * < 0.05, ** < 0.01, *** < 0.001.

### 3.4 Body composition and blood parameter shifts

**Figure 3:**
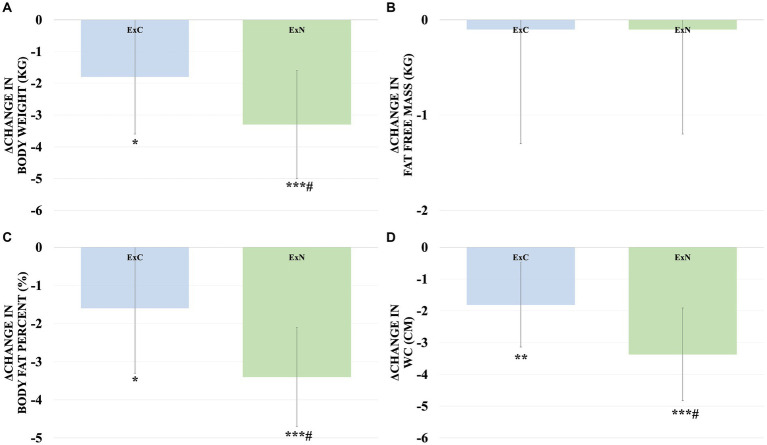
Comparison of changes in body composition components for ExC group and ExN group before and after participating in the intervention program. **(A)** Changes in body weight. **(B)** Changes in fat free mass. **(C)** Changes in body fat percent. **(D)** Changes in WC. Values are mean ± SD, * <0.05 vs. baseline, ** <0.01 vs. baseline, *** <0.001 vs. baseline, # <0.05 vs. ExC.

**Figure 4:**
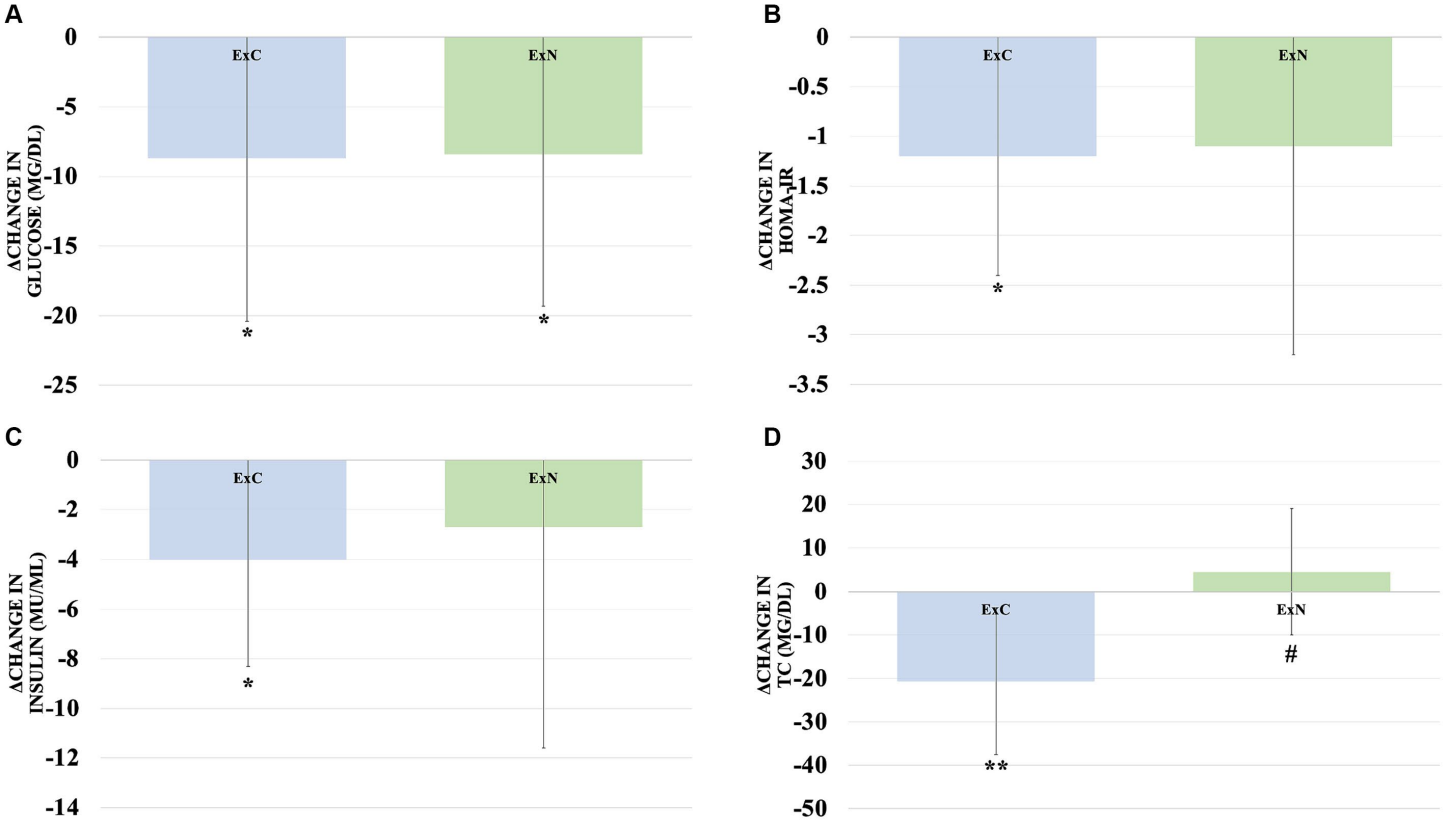
Comparison of changes in body glucose metabolism biomarkers for ExC group and ExN group before and after participating in the intervention program. **(A)** Changes in glucose. **(B)** Changes in HOMA-IR. **(C)** Changes in insulin. **(D)** Changes in TC. Values are mean ± SD, * <0.05 vs. baseline, ** <0.01 vs. baseline, *** <0.001 vs. baseline, # <0.05 vs. ExC.

Throughout the study, both groups exhibited improvements in obesity-related body composition measures. Notably, the ExN group displayed remarkable enhancements, including a substantial reduction in body weight (5.2%, *p* < 0.001), a significant decrease in body fat percentage (9.5%, *p* < 0.001), and a considerable reduction in waist circumference (10.7%, *p* < 0.001) compared to the ExC group. Shifting our focus to obesity-related blood variables, significant reductions were observed in the ExC group for total cholesterol (TC) (10.9%, *p* < 0.001), high-density lipoprotein (HDL) (12.1%, *p* < 0.05), glucose (9.2%, *p* < 0.05), insulin (30.5%, *p* < 0.05), and the Homeostatic Model Assessment for Insulin Resistance (HOMA-IR) (38.7%, *p* < 0.05). Similarly, while the ExN group exhibited a significant decrease in glucose (8.5%, *p* < 0.05). To aid in understanding these results, Figures 3, 4 visually depict changes in body composition parameters and glucose metabolism biomarkers before and after the intervention for both groups. In Figure 3, both groups exhibited significant decreases in body weight (ExC: *p* < 0.05, ExN: *p* < 0.001), with the ExN group showing a significant decrease than the ExC group (*p* < 0.05). Similarly, both groups demonstrated significant decreases in body fat percentage (ExC: *p* < 0.05, ExN: *p* < 0.001), with the ExN group displaying a significant decrease than the ExC group (*p* < 0.05). Noteworthy decreases in WC were observed in both groups (ExC: *p* < 0.01, ExN: *p* < 0.001), and the ExN group exhibited a significant decrease than the ExC group (*p* < 0.05). In Figure 4, both the ExC and ExN groups exhibited significant decreases in glucose (*p* < 0.05), with the ExC group also showing significant decreases in HOMA-IR and Insulin (*p* < 0.05). Additionally, for TC, the ExC group demonstrated a significant decrease (*p* < 0.01) compared to baseline, while the ExN group displayed a notably lower value than the ExC group (*p* < 0.05). These collective results emphasize the substantial and differential impacts of the intervention on health-related parameters, suggesting the potential for meaningful improvements in both body composition and metabolic health (Table 4).

**Table 4:** Baseline and follow-up characteristics of the subjects.

Variables	Exercise and control (*n* = 10) Age: 28.3 ± 4.3								Exercise and nutrition (*n* = 11) Age: 32.0 ± 5.3									
	Week 0		Week 4		Week 8		*d*		Week 0		Week 4		Week 8		*d*		P*	P†
	*M*	SD	*M*	SD	*M*	SD	*M*	SD	*M*	SD	*M*	SD	*M*	SD	*M*	SD		
**Body composition**																		
Body weight (kg)	67.1	10.7	65.8	10.2	65.4	10.3	−1.8*	1.8	64.1	9.6	62.0	8.8	60.8	8.9	−3.3***	1.7	0.506	0.048*
Body fat mass (kg)	23.8	6.9	22.9	6.7	22.1	6.4	−1.7**	1.6	23.3	6.7	21.5	5.7	20.1	6.3	−2.7***	1.5	0.878	0.013*
Fat free mass (kg)	43.3	4.8	43.0	5.1	43.3	5.0	−0.1	1.2	40.8	4.8	40.5	4.4	40.7	4.4	−0.1	1.1	0.239	0.956
BMI	24.9	3.8	24.4	3.7	24.0	3.6	−0.8**	0.8	24.3	3.8	23.5	3.6	22.9	3.5	−1.4***	0.7	0.736	0.097
Percent body fat (%)	34.8	5.6	34.2	6.2	33.2	5.7	−1.6*	1.7	35.8	6.6	34.2	5.4	32.4	6.9	−3.4***	1.3	0.714	0.011*
WC (inch)	31.9	3.7	32.0	3.8	30.1	3.5	−1.8**	1.3	31.8	3.3	29.8	3.2	28.4	2.9	−3.4***	1.5	0.961	0.020*
**Glucose metabolism biomarkers**																		
HDL (mg/dL)	64.2	6.7			56.4	6.6	−7.9*	9.0	63.4	12.5			62.4	12.5	−1.3	8.8	0.929	0.109
Glucose (mg/dL)	93.3	6.5			84.7	9.6	−8.7*	11.7	98.9	14.3			90.5	10.9	−8.4*	10.9	0.273	0.962
TC (mg/dL)	189.5	22.9			168.8	21.1	−20.7**	16.8	190.5	25.0			195.0	23.5	4.5	14.5	0.925	0.002**
Insulin (μU/mL)	13.1	4.5			9.1	4.0	−4.0*	4.3	14.3	8.8			11.6	8.3	−2.7	8.9	0.682	0.678
HOMA-IR	3.1	1.2			1.9	0.8	−1.2*	1.2	3.6	2.5			2.5	1.5	−1.1	2.1	0.542	0.867
ALTGPT (U/L)	11.5	2.9			9.6	2.3	−1.9*	2.1	13.4	5.8			12.2	2.8	−1.1	5.3	0.361	0.696
ASTGOT (U/L)	15.5	2.6			15.5	3.2	−0.1	2.8	16.5	2.2			16.0	2.5	−0.5	1.9	0.352	0.668
Uric acid (mg/dL)	4.8	0.7			4.9	1.3	0.1	1.3	4.8	1.3			4.8	1.1	0.0	0.8	0.973	0.911
Total OC (nmol/L)	18.7	5.1			17.8	4.7	−0.9	4.4	16.1	4.1			16.6	4.8	0.5	2.8	0.200	0.397
COC (nmol/L)	14.4	4.0			13.6	4.9	−0.8	3.4	12.5	3.7			12.5	4.3	0.0	3.1	0.293	0.593
UCOC (nmol/L)	4.4	2.9			4.3	2.6	−0.1	1.4	3.5	2.0			4.0	2.7	0.5	1.7	0.447	0.378
TG (mg/dL)	69.9	21.3			67.2	21.7	−2.7	23.8	108.2	102.2			91.0	47.9	−17.1	65.3	0.260	0.517

M, mean; SD, standard deviation. Week 0, before intervention; week 4, at the beginning of the 4th week of the intervention; week 8, after intervention; d: (week 8) – (week 0). P difference between baseline and follow-up values; * < 0.05, ** < 0.01, *** < 0.001, P* difference between baseline measures between the groups; P† difference in changes in the measures between the groups. BMI, body mass index; WC, waist circumference; SBP, systolic blood pressure; DBP, diastolic blood pressure; TC, total cholesterol; HDL, high-density lipoprotein cholesterol; TG, triglyceride; HOMA-IR, homeostatic model assessment for insulin resistance; OC, osteocalcin; COC, carboxylated osteocalcin; UCOC, undercarboxylated osteocalcin.

## 4 Discussion

Sugar restriction, characterized by the limitation of sugar-rich snacks and beverages over an 8-week program and combined with non-face-to-face brisk walking exercise, led to additional improvements in body composition parameters. These parameters included body weight, body fat mass, percent body fat, and waist circumference, in comparison to the results seen in the group that only engaged in brisk walking. These findings suggest that the combination of nutritional intervention and exercise proves effective for weight management in young overweight women aged 20–39 living alone. However, contrary to our primary hypothesis, the nutritional intervention did not result in additional enhancements in glucose metabolism biomarkers.

A substantial body of research consistently suggests that brisk walking presents a viable strategy for promoting weight loss among young women in their 20s and 30s. Additionally, emerging evidence indicates that brisk walking may have favorable effects on lipid profiles (20). In this study, the exercise and control group experienced significant reductions in key measures, including body weight (1.8 ± 1.8 kg), and percent body fat (1.6 ± 1.7%). These findings align with our initial hypothesis and are in line with previous research outcomes. In a sedentary population of overweight women aged 21–30, engaging in in-person brisk walking for 45 min, five times a week, over 10 weeks led to significant weight reduction (17). Similarly, among sedentary young adults aged 20–40 who participated in 30 min of daily in-person walking exercise for 8 weeks, there was a notable reduction in percent body fat by (*p* < 0.05) (36).

Furthermore, physical activity represents a vital lifestyle intervention that has demonstrated its efficacy in improving glucose metabolism and reducing the risk of overweight. In this study, the exercise and control group exhibited significant decreases in fasting glucose by 9.2%, insulin by 30.5%, and HOMA-IR by 38.7%. These findings are consistent with prior research, confirming the favorable impact of exercise interventions on glucose metabolism biomarkers. According to Sandvei et al. (37), young adults engaging in continuous moderate-intensity aerobic training three times a week for 8 weeks at an intensity of 70–80% of maximal heart rate experienced a significant reduction in fasting glucose levels by 3.6%. Moreover, in obese young adults, insulin levels decreased by 45.4% following the eight-week aerobic exercise program (38). Remarkably, even though brisk walking was conducted in a non-face-to-face manner over 8 weeks in this study when compared to previous studies conducted in a face-to-face setting, it was observed that fasting glucose levels decreased to a greater extent, and insulin levels also decreased significantly, albeit not to the same extent as in the face-to-face program. This suggests that in the future, leveraging advanced devices and technologies for non-face-to-face brisk walking exercises could be an effective approach for improving glucose metabolism biomarkers in young obese women.

Our 8-week program, focusing on restricting sugar-rich snacks and beverages to reduce carbohydrate intake, resulted in notable improvements in body composition. While both groups experienced significant decreases in various body composition parameters, it is crucial to emphasize that the exercise and nutrition group achieved substantially greater reductions compared to the exercise and control group. Specifically, the exercise and nutrition group exhibited significant decreases in body weight, body fat mass, percent body fat, and waist circumference. In contrast, the exercise and control group showed reductions of a lesser magnitude, including decreases in body weight, body fat mass, percent body fat, and waist circumference. Previous reviews underscore the importance of reducing sugar-rich snack and beverage consumption as a complementary strategy for maintaining a healthy weight and promoting overall healthy dietary patterns (14, 15). A meta-analysis revealed that substituting sugar-sweetened beverages with low- or no-calorie sweetened alternatives was associated with weight reductions with a mean difference of −1.06 (−1.71 to −0.41 kg) (39), aligning with our study’s observation of a significantly greater reduction in body weight in the exercise and nutrition group that restricted their consumption of sugar-rich snacks and beverages compared to the exercise and control group. From a public health perspective, the integration of brisk walking with the reduction of sugar-rich snack and beverage consumption emerges as a straightforward and effective approach to facilitate weight loss and enhance overall well-being. Nevertheless, the widespread implementation of these interventions may necessitate policy modifications or environmental enhancements, including the provision of safe and walkable spaces or the implementation of taxes on sugar-sweetened beverages (40).

The exercise and nutrition group exhibited a notable reduction in fasting glucose levels (8.4 ± 10.9 mg/dL) following the 8-week intervention. However, no significant difference in the changes in fasting glucose was observed when compared to the exercise and control group. These outcomes suggest that there was no significant additional effect on glucose metabolism biomarkers when combining an exercise program with a nutrition program. Notably, the participants in this study did not display markedly elevated values for diabetes-related variables, particularly fasting glucose (98.9 ± 14.3 mg/dL) before the intervention. Therefore, despite the evident effectiveness of limiting the consumption of sugar-rich snacks and beverages in improving body composition, no substantial changes were observed in glucose metabolism. Moreover, it is important to acknowledge that the intervention period lasted only 8 weeks, which may have been insufficient to induce significant alterations in glycemic control indicators and other metabolic health parameters, especially among individuals whose initial glycemic profiles fell within the normal physiological range. Furthermore, lifestyle factors can exert a significant influence on glucose metabolism. One possible contributing factor is the timing and frequency of meals and snacks within individuals’ daily routines. In the case of single-person households in Korea, dining alone is common due to living arrangements, resulting in inconsistent mealtime and eating patterns (41).

In addition, while our study highlights the benefits of brisk walking combined with nutritional interventions for improving body composition and glucose metabolism, it is important to acknowledge the potential of other exercise forms. Resistance training, for example, has been demonstrated to also offer significant benefits in the improvement of glucose metabolism and body composition (42, 43). This suggests that integrating diverse forms of physical activity could further enhance the efficacy of lifestyles interventions targeted at metabolic health improvements. Such findings advocate for a broader inclusion of varied exercise modalities in future studies, potentially offering a more comprehensive approach to managing and improving metabolic health in similar populations.

The current study is subject to several limitations. Firstly, we did not implement controls to monitor participants’ sleep patterns or collect corresponding sleep-related data. Sleep quality and duration significantly impact various aspects of health, including glucose metabolism and overall energy levels. Individual variations in sleep patterns, such as inadequate sleep, irregular sleep schedules, or sleep disruptions, could introduce confounding variables influencing the study’s outcomes. The second limitation of our study is the small sample size, which was significantly influenced by external factors beyond our control, primarily the COVID-19 pandemic. The pandemic and the consequent social distancing measures imposed severe recruitment challenges. Many potential participants were hesitant to engage in a study involving physical visits during this period. Furthermore, there were several instances of participant dropouts due to COVID-19 infections and related concerns, which compounded the difficulty of maintaining a larger, stable cohort. To address this limitation and enhance the robustness of future studies, we recommend adopting more flexible and resilient recruitment and data collection methods that can adapt to similar disruptions. Thirdly, our examination of participants’ carbohydrate intake, as presented in Table 3, did not specifically measure sugar intake. In this study, an analysis of sugar intake was excluded from our assessment due to limitations in the nutrient analysis program used. Although this limitation prevents us from offering comprehensive insights into sugar consumption patterns, we believe that our study still provides valuable insights into other dietary components. Notably, the ExN group showed a significant reduction in carbohydrate intake between the week 0 and week 8, which includes sugars, suggesting that the observed effects could be partially attributed to reduced sugar consumption. While we cannot directly attribute the effects to sugar alone, the reduction in total carbohydrate intake likely reflects a decrease in sugar-rich foods. Future studies should include more detailed dietary assessments to differentiate the specific contributions of sugar reduction versus overall carbohydrate reduction This distinction is essential for developing more targeted and effective dietary strategies for weight management in young overweight women.

Our results indicate that a program focused on restricting the consumption of sugar-rich snacks and beverages may serve as a viable approach within diet-exercise strategies to promote weight loss among young women who are overweight or obese. These findings underscore the promise of implementing non-face-to-face nutrition and exercise behavioral programs in clinical practice, aiming to address overweight in young adult women living in single-person households. Future research is warranted to validate our observations, explore the long-term effects, and assess the feasibility of these non-face-to-face interventions by employing extended intervention durations.

## Data availability statement

The raw data supporting the conclusions of this article will be made available by the authors, without undue reservation.

## Ethics statement

The studies involving humans were approved by Korea University Institutional Review Board of the Korea (reference number: KUIRB-2021-0146-01). The studies were conducted in accordance with the local legislation and institutional requirements. The participants provided their written informed consent to participate in this study.

## Author contributions

YL: Conceptualization, Data curation, Formal analysis, Investigation, Methodology, Writing – original draft, Writing – review & editing. NK: Data curation, Formal analysis, Software, Validation, Visualization, Writing – review & editing. SG: Data curation, Investigation, Methodology, Writing – review & editing. JK: Conceptualization, Funding acquisition, Project administration, Resources, Supervision, Writing – review & editing. JP: Conceptualization, Funding acquisition, Project administration, Resources, Supervision, Validation, Writing – review & editing.
